# Development of 3D-Printed Chewable Gummy Tablets with Adjustable Ondansetron Content for the Treatment of Pediatric Patients

**DOI:** 10.3390/pharmaceutics17040458

**Published:** 2025-04-02

**Authors:** Martin Veselý, David Záruba, Jan Elbl

**Affiliations:** Department of Pharmaceutical Technology, Faculty of Pharmacy, Masaryk University Brno, Palackého Tř. 1946/1, 612 42 Brno, Czech Republic

**Keywords:** 3D print, SSE, individualized therapy, gummy formulation, ondansetron

## Abstract

**Background/Objectives**: Semi-solid extrusion (SSE) 3D printing is an innovative method utilized for preparation of various drug dosage forms, allowing for individualization by means of incorporation of one or multiple drugs in adjustable doses. SSE provides repeatable results and can be conveniently utilized in small batch production. This study aimed to develop a chewable formulation for pediatric patients which could be easily printed using SSE. **Methods**: Pectin and gelatin were utilized as gel-forming agents, polyvinylpyrrolidone as a thickener, glycerol as a plasticizer, citric acid as a pH modifier, and potassium sorbate as a conserving agent. Obtained tablets were evaluated for mass and content homogeneity and their mechanical properties compared to the long-time market standard for gummies. **Results**: Gummy formulation with texture properties comparable to the selected standard and mass homogeneity were prepared. The linear correlation between the model size and ondansetron content was proven. **Conclusions**: SSE 3D printing thus presents a suitable method of gummy formulation production with possible adjustment of dose by defining the object size.

## 1. Introduction

Three-dimensional printing is an innovative technique widely utilized in pharmaceutical research. Its principle is based on gradual deposition of material in a layer-by-layer manner [1,2]. This technology enables the fabrication of products with customizable size, color or flavor, significantly contributing to manufacturing individualization and allowing precise drug dosing [3,4]. Such personalization is mostly beneficial for patients with pharmacokinetic profiles that deviate from the general population, namely, pediatric or geriatric patients, as well as for treatments involving medicines with a narrow therapeutic window [5,6]. Moreover, 3D printing facilitates the preparation of dosage forms containing drug combinations that are not commercially available, hence simplifying the dosing regimen [7].

For a long time, pediatric patients were off the sight of pharmaceutical research. Usually, tailoring the dose of medicines designated for adults is implemented in their treatments, which is not always appropriate, as pharmacokinetic differences extend beyond body weight considerations [8,9,10]. Pediatric patients undergo rapid physiological and biochemical changes, resulting in significant variability in pharmacokinetics and forming a highly heterogenous group of patients [11,12]. Three-dimensional printing offers a promising solution by enabling a high level of dose individualization, allowing personalized dosing based on factors such as age and weight [13]. A very common challenge in pediatric therapy is poor patient compliance, often due to swallowing difficulties of certain dosage forms or aversion to unpleasant taste [14]. Several strategies to address this issue, such as incorporation of cyclodextrins [15,16], sweeteners and flavors [17], and printing dosage forms of enticing shape, are already documented [18]. Another example of increasing the compliance of pediatric patients is the administration of chewable gummy tablets [19]. Designed to be chewed and then swallowed, these dosage forms benefit patients with dysphagia or those who often struggle to swallow conventional dosage forms such as tablets or capsules. Notably, 1 in 11 primary care patients report frequent difficulties with swallowing medicines [20]. Additionally, chewable gummy tablets allow for higher drug loads, as they eliminate the need to swallow large-volume dosage forms in a single unit. However, their higher water content may reduce shelf life. Furthermore, due to prolonged interaction (compared to other peroral dosage forms) between the chewable gummy tablet formulation and taste buds, effective taste-masking strategies such as the use of sweeteners and flavoring agents are essential [21,22]. To enhance their acceptability among pediatric patients, chewable gummy tablets should possess a pleasant taste, texture, and appearance to encourage their adherence to treatment [23]. Research in this area primarily focuses on gel chewable tablets, which share similarities with conventional tablets but comprise also some gel-forming excipient [24]. Utilization of pectin [25], gelatin [19], alginates, hyaluronic acid, and cellulose derivates is documented [17].

Conventionally chewable tablets are manufactured by direct compression, a cost-effective and straightforward process. This method eliminates the need for using high temperatures, lowering the risk of decomposition of certain temperature labile medicines. Another benefit is the absence of moisture, resulting in lower occurrence of microbial contamination. However, direct compression has certain limitations, including constraints on the drug load due to potential adverse effects on tablet properties. Moreover, direct compression of some ingredients may be unfeasible [26].

These challenges can be mitigated with the use of SSE 3D printing, as this method typically operates at low temperatures, minimizing the risk of medicine degradation [27]. While SSE is relatively cost-effective, it remains more expensive than conventional manufacturing techniques. The process utilizes pre-filled syringes containing pastes or gels, with possible addition of an active drug ingredient [28]. This setting furthermore meets the critical quality attributes set by regulatory agencies, simplifying potential introduction of the printed medicines to the market. Ink preparation is mostly considered straightforward and time-efficient [1]. By modifying the model shape or size, various dosage forms can be printed to meet specific patient or clinician needs, making it possible to produce higher-dose formulations compared to direct compression [29,30]. One of the limiting factors is printability, which highly depends on the viscoelastic properties of the printed mixture. The range of materials suitable for SSE 3D print is relatively limited, as many are susceptible to degradation under high shear stress conditions. To ensure proper flow of material through the nozzle, applied pressure must induce a significant decrease in G′ (storage modulus, representing solid-like behavior) relative to G″ (loss modulus representing liquid-like behavior). Upon pressure release, G′ should recover to its original value, demonstrating the solid state of the printed objects [31,32]. This solidification process takes time as the object must solidify sufficiently, making the process more time-challenging than other conventional ways of production such as direct compression. The final product properties such as hardness might also be compromised [27]. The overall knowledge of requirements on the viscoelastic properties remains limited and research in this area is largely based on trial and error [33]. Nevertheless, achieving the necessary viscoelastic properties is essential to ensuring the successful printing of dosage forms that deliver precise drug doses.

Ondansetron is a cost-effective and widely used medicine in the treatment of pediatric patients that experience nausea and vomiting from gastroenteritis after chemotherapy, radiotherapy, or after concussion or other kinds of head trauma. These symptoms affect at least 25% of patients and are among the most frequently reported complications. Ondansetron is well accepted and reduces the rates of return visits [34,35,36,37]. Due to its selective antagonist effect on 5-hydroxytryptamine 3 (5-HT_3_) receptors, it does not induce extrapyramidal side effects, making it a suitable option for pediatric treatment [38]. Additionally, ondansetron plays a role in preventing complications such as dehydration, hypokalemia, and aspiration [39]. A common challenge in nausea management is the inability to swallow solid dosage forms, such as solid tablets or capsules. Consequently, ondansetron is often administered as orodispersible films or tablets, which do not require water for ingestion—an advantage, as consuming water may trigger vomiting and counteract the therapeutic effect [40]. There are a limited number of 3D printing studies focusing on ondansetron containing dosage forms. Successful printing of orodispersible tablets by selective laser sintering technique was recorded though [41]. To the best of our knowledge, there is no commercially available ondansetron chewable gummy tablet on the market (see Appendix A Table A1 for the summary of commercially available ondansetron-containing drug dosage forms for pediatric patients) and no study on SSE 3D printing of chewable gummy tablets with the content of ondansetron has ever been conducted.

Thus, the aim of this study is to develop chewable gummy tablets containing ondansetron as an alternative administration method. The study focused on achieving a 4 mg dose, which is commonly administered three times daily in clinical practice [42]. This widely prescribed dose serves as a reference point from which customized doses could be derived. As the primary objective was to assess the feasibility of SSE 3D printing for this formulation, it was assumed that once a 4 mg tablet was successfully printed, the process could be adapted to different pediatric doses by simply modifying the tablet size. It was further evaluated whether the formulations exhibit an immediate release of ondansetron. The study began with the formulation of the printing mixture, testing different ratios of pectin to gelatin to optimize its composition. Subsequently, the effects of addition of sweetener, antimicrobial substance, coloring, and flavoring agents were evaluated. After optimizing the formulation, print repeatability was assessed before incorporating ondansetron hydrochloride dihydrate. The next phase involved examining the influence of air pressure on the tablet’s average weight and physical appearance, followed by optimization. Finally, the correlation between the 3D model dimensions and ondansetron content was analyzed to ensure accurate dosing.

## 2. Materials and Methods

### 2.1. Materials

**Figure 1:**
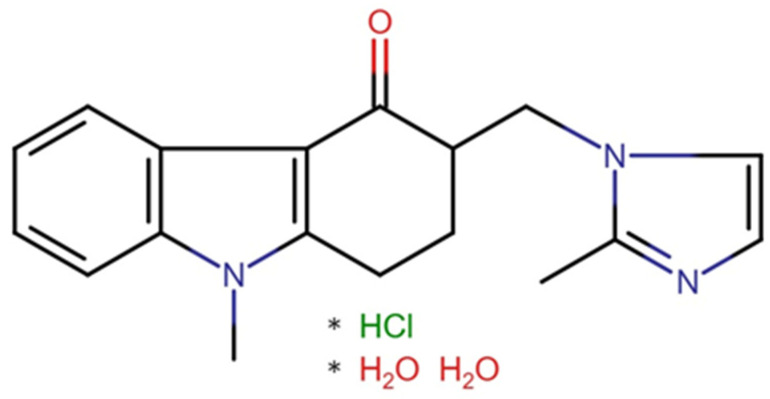
The chemical formula of ondansetron hydrochloride dihydrate (* stands for the chemical bond).

Ondansetron hydrochloride dihydrate (O) (Qufu Hongly Chemical Industry Co., Ltd., Yangzhuang Lingcheng Town, Qufu, China) was used as the active ingredient (presented in Figure 1). Pectin from apple (P) (Sigma Aldrich, Taufkirchen, Germany) and gelatin (G) (Dr. Kulich Pharma, Hradec Králové, Czechia) acted as gelling agents. Polyvinylpyrrolidone (PVP) (Sigma Aldrich, Taufkirchen, Germany) was used as a viscosity altering agent, potassium sorbate (PS) (Sigma Aldrich, Taufkirchen, Germany) played the role of an antimicrobial substance, saccharose (S) (Cukrovar Vrbátky, Vrbátky, Czechia) served as a sweetening agent, citric acid (CA) (Dr. Kulich Pharma, Hradec Králové, Czechia) as a pH and flavor-altering agent, and glycerol (GLY) (Dr. Kulich Pharma, Hradec Králové, Czechia) as a plasticizer. Orange and yellow colorants were purchased at Aromka Brno, Brno, Czechia. Purple colorant was purchased at Zan-aromi, Brno, Czechia. Raspberry, cherry, and punch flavoring agents were purchased at Aroco, Prague, Czechia. Purified water (W) with a quality corresponding to Ph. Eur. 10.0, Chapter 5. was used [43].

Haribo^®^ gummy bears, marketed since 1922, and thus being a very well-known gummy formulation, were used as a reference sample by texture characterization and moisture content. Their composition is as follows: glucose syrup, sugar, gelatin, dextrose, fruit juices from concentrates (apple, raspberry, strawberry, orange, lemon, pineapple), citric acid, sunflower oil, fruit and plant concentrates (dyer’s flare, apple, spirulina, blackcurrant, kiwi, lemon, mango, and others), beeswax, artificial flavors.

### 2.2. Print Dispersion Preparation

**Figure A1:**
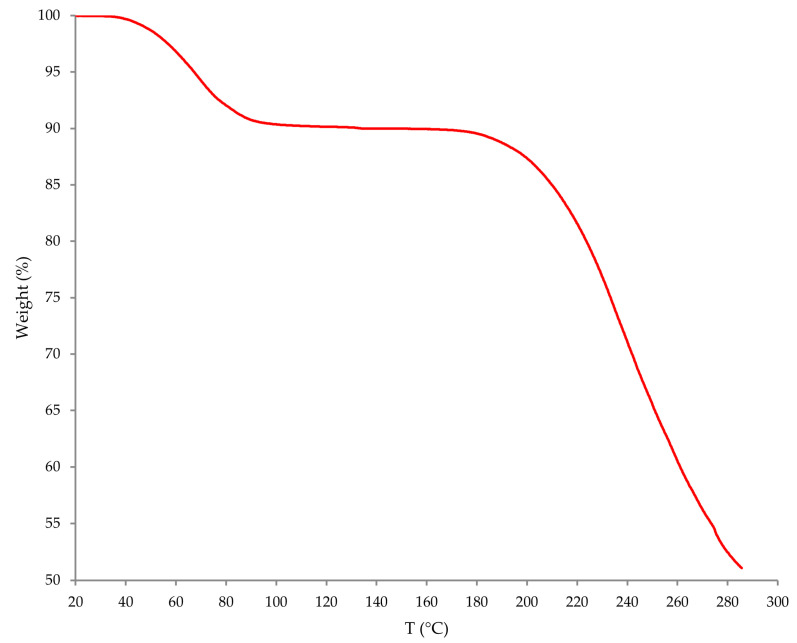
TGA spectrum of ondansetron hydrochloride dihydrate.

To prepare the printing material, gelatin was spread on the surface of half the amount of water needed, left to swell for 15 min, and dissolved over the water bath (65 °C) afterwards. Concurrently, the pectin was separately dispersed into glycerol, while the rest of the ingredients were gradually dissolved in water. Saccharose was added in the form of simple syrup (64 wt.% of saccharose and 36 wt.% of purified water) prepared according to the European Paediatric Formulary [44]. Prior to ondansetron hydrochloride dihydrate incorporation, its solvatomorphic form was confirmed utilizing thermogravimetric analysis (TGA; see Appendix B Figure A1). All mixtures were slowly mixed while preheated to and kept at 45 °C. Maintaining the constant temperature was a critical factor in formulating the mixture intended for gummy formulation printing. The colorant and flavoring agents were added as a final step. The container, covered by plastic protective film to prevent the evaporation of water, was left in a water bath set at 45 °C for 10 min. All print dispersion types are described in Table 1. The excipients were chosen primarily for their cost-effectiveness and widespread availability.

### 2.3. SSE 3D Printing

Digital models of chewable gummy tablets were prepared in Autodesk Inventor 2022.4.1 (Autodesk, San Francisco, California, USA) CAD software. Each batch consisted of 20 cylindrical tablets having 10 mm diameter (the base model) and a height of 4.5 mm (5 layers per 0.9 mm layer height). This design was saved as a stereolithographic file (.stl) and imported to PrusaSlicer 2.8.1 (Prusa Research, Prague, Czechia).

**Figure 2:**
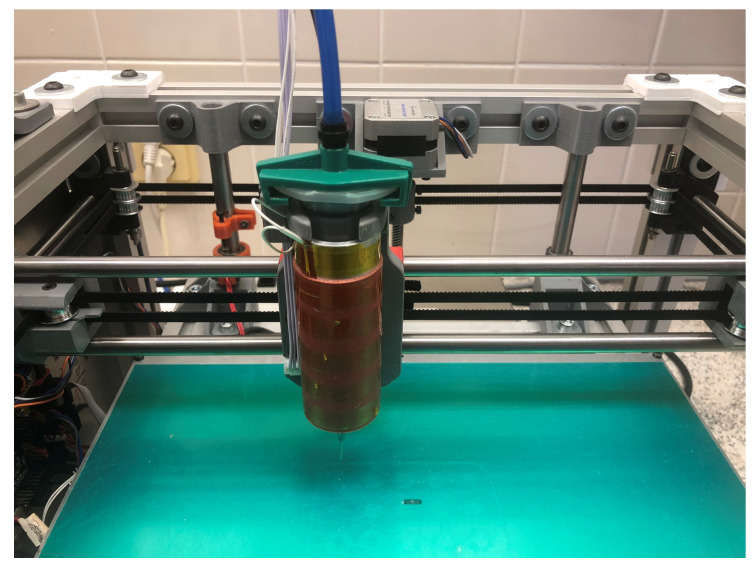
SSE 3D printer utilizing pressured air (blue tubing) and a heating sleeve.

For the actual printing, an in-house modified SSE 3D (presented in Figure 2) printer utilizing compressed air was used. Polypropylene syringes of 30 mL volume (Hot Air, Ostrava, Czechia) were employed, and material was extruded through an 18 G stainless needle tip (0.84 mm inner diameter). A heating sleeve surrounding the syringe maintained the printing mixture at 45 °C, enabling its extrusion under optimal air pressure. Tablets were printed on 90 µm thick polyester masking tape (Lepíky Ltd., Prague, Czechia) laid on a 2 mm glass sheet.

The print settings were as follows: print speed 20 mm/s, extrusion width 0.84 mm, 1 perimeter, and 100% rectilinear pattern infill density. The tablets were left in the fridge for 10 min to reduce their stickiness before evaluation.

### 2.4. Mass Uniformity Testing

All 20 tablets were manually weighed using KERN 220-4N analytical scales (Gottl. KERN & Sohn GmbH, Balingen-Frommern, Germany). The mass uniformity test was conducted afterwards.

### 2.5. Residual Moisture Measurement

Three randomly selected samples weighing at least 1 g were evaluated using the moisture analyzer Excellence Plus HX204 (Mettler Toledo, Columbus, OH, USA). The temperature was set to 105 °C and sample evaluated until the mass change was lower than 1 mg within 50 s. The presented values are in %.

### 2.6. Texture Profile Analysis

In terms of texture properties, the hardness (referred to as resistance against compression) and springiness index were evaluated. For this purpose, a CT3 Texture Analyzer (AMETEK Brookfield, Middleborough, MA, USA) equipped with a 4.5 kg load cell and controlled by TexturePro CT V1.8 software (AMETEK Brookfield, Middleborough, MA, USA) was used. For the test, 5 randomly selected samples were chosen. The tablet was placed on a plain surface of a metal plate while a cylindrical probe TA-11 was moving down at a speed of 0.5 mm/s compressing the sample by 2 mm in 2 cycles (same distance for both cycles). The trigger load was set to 0.098 N (10 g) in both cycles.

### 2.7. Disintegration Time Measurement

Apparatus A described in Ph. Eur. 10.0, Chapter 2.9.1. made up of six glass tubes with the mesh screen at the bottom was used to evaluate the disintegration time [45]. Six randomly selected gummy chewable tablets were selected for the measurement. As the worst-case scenario of swallowing the tablet in one piece was considered, 700 mL solution of simulated gastric fluid (corresponding to pH of 1.2) was used to simulate the gastric environment. The testing temperature was set to 37 °C. The samples were cyclically immersed and withdrawn from the solution (30 cycles per minute), and the disintegration time was visually confirmed by the complete absence of any visible tablet remnants.

### 2.8. Dissolution Test

The dissolution method was adopted from Tagami et al. (2021) and slightly modified to fit our requirements [19]. A Sotax AT7 off-line dissolution apparatus (Sotax AG, Aesch, Switzerland) in paddle setup was employed, where six randomly selected intact tablets were evaluated. The dissolution vessels were filled with 900 mL of simulated gastric fluid solution (pH 1.2) and stirred at 50 rpm at 37 °C. The sample of 1 mL was collected at following time points: 5, 10, 15, 30, 45, 60, 90, and 120 min. The course of dissolution was observed and pictures were taken. The ondansetron content in samples was measured utilizing the HPLC method described below.

### 2.9. High Performance Liquid Chromatography

Ondansetron hydrochloride dihydrate content was measured in 10 tablets and recalculated to pure ondansetron afterwards. Each tablet was dissolved in 100 mL of purified water, heated to 45 °C for 10 min to speed up the process. To provide sufficient dissolution, the flasks were left overnight. Samples were then filtered through filters (Labstore, Prague, Czechia) having 0.45 µm pore size and the content was measured using HPLC (Agilent Technologies, Santa Clara, CA, USA).

The content assay method was adopted from Kowtharapu et al. (2022) with slight modifications to suit our experimental requirements [46]. Mobile phase of acetonitrile and a 20 mM solution of phosphoric acid in the 15:85 (*v*/*v*) ratio were employed at a flow rate of 1 mL/min, yielding pressure of about 100 bar. A Nucleodur 100-5 CN column maintained at 30 °C was used. The injection volume was set to 5 µL and the eluent was screened at a wavelength of 310.4 nm. The retention time was approximately 5 min.

The HPLC method was validated by analyzing the calibration solutions of ondansetron hydrochloride dihydrate. A total of eight solutions, with concentration ranging from 0.01 to 0.08 mg/mL (recalculation to the ondansetron base), were prepared to encompass the expected concentration range of samples (0.02–0.06 mg/mL). Calibration and standard measurements were repeated after one week to confirm the stability of prepared samples.

## 3. Results and Discussion

### 3.1. Three-Dimensional Printing Process

During the initial printing (non-published data), dispersions containing either pectin or gelatin as a gel agent were tested. It was observed that the pectin significantly improved the structural integrity of the tablets, while gelatin enhanced their elasticity. Additionally, the inclusion of polyvinylpyrrolidone appeared crucial as the tablets printed without it exhibited poor shape retention. Consequently, polyvinylpyrrolidone concentration was maintained at a fixed level, and the focus shifted to optimizing the ratio of pectin to gelatin. Gelatin is widely used in the production of gummy formulations, although nowadays it is extensively substituted by modified starches. It was also utilized in the study made by Herrada-Manchón et al. (2020), who employed the SSE 3D printing method to produce gummy formulation with ranitidine content. As well as gelatin, corn starch, xanthan gum, and carrageenan were involved as gel agents to successfully produce three differently shaped gummies with low heterogeneity, ruptures, and imperfections present [47]. In our study, similar results were achieved as all samples were successfully printed exhibiting good shape retention, elasticity, and a smooth surface. The tablets remained stable upon handling and showed no signs of disintegration.

The only exception to the otherwise satisfactory tablet appearance was observed in samples printed at higher air pressures, which were produced to investigate their influence on tablet mass and uniformity. These samples exhibited significant surface irregularities, including noticeable protrusions, resulting in an unsatisfactory appearance due to material over-extrusion.

**Figure 3:**
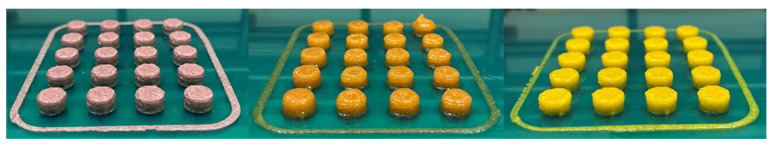
Three-dimensional-printed gummy formulation with the addition of colorants.

To enhance the visual appeal of the tablets, the incorporation of certain coloring agents was explored. This approach proved effective in improving tablet aesthetics and could potentially contribute to better compliance among pediatric patients, as previously discussed. The final printed tablets are presented in Figure 3.

The initial step involved determining the optimal ratio of pectin to gelatin, for which 5 different ratios were tested. After finding the optimal ratio, potassium sorbate and simple syrup were incorporated, and their addition was evaluated while assessing process repeatability by printing 3 batches with identical composition. In the final step, ondansetron hydrochloride dihydrate was incorporated, and its solvatomorphic form was verified via TGA. Potential incompatibilities between ondansetron hydrochloride and other components were evaluated based on the available literature. The analysis indicated that none of the ingredients was expected to be incompatible with ondansetron hydrochloride, as most are commonly used in pharmaceutical formulations and exhibit minimal incompatibility [48,49]. The primary concern was the formation of precipitates; however, no precipitation occurred during the process. Upon the addition of the active substance, the effect of varying air pressures on mass of tablets and its uniformity was also examined. The composition of all print dispersion is stated in Table 1.

### 3.2. Mass Uniformity

During the optimization phase, two batches with the highest pectin content (P6.5G9 and P7G8) exhibited a substantial number of samples outside the acceptable weight variation limits specified in Ph. Eur. 10.0, Chapter 2.9.5 (±5% from the average) [45]. Given that such deviations could impact the active substance content, these batches were excluded from further evaluation. Repeatability testing of the P6G10 batch with added colorants, flavoring agents, sweetener, and antimicrobial substance demonstrated satisfactory results as all samples complied with the specified limits. Furthermore, all batches containing ondansetron hydrochloride dihydrate were within the specified limits, indicating promising uniformity in ondansetron content. A strong correlation between the 3D model volume (50, 75, 100, 125, and 150% of the original model size) and the corresponding tablet weight was observed, confirming that model size has an outright influence on the object weight. The results obtained are presented in Table 2. To potentially enhance the efficiency of the weighting process, integrating a balance into the printing platform could be considered. Such an approach has been previously documented and demonstrated as an effective in-line control mechanism [50].

### 3.3. Evaluating the Air Pressure Influence

The value of air pressure utilized in SSE 3D printing mainly influences the average mass of the printed tablets as it determines the amount of material that is pushed through the nozzle per unit of time. Since exaggerated setting of the pressure can lead to over-extrusion, the impact on the tablet appearance may also be significant. Therefore, we decided to investigate these two parameters by printing the same composition tablets using different air pressures, namely, 55, 65, 75, and 85 kPa.

The significant impact on appearance was clearly visible. The higher the air pressure, the worse looking tablets were produced due to over-extrusion. The imperfections mostly included uneven surface or bulky looking tablets. The appearance of the samples printed at 55 kPa was also unsatisfactory. It was therefore decided to keep the air pressure at the original 70 kPa.

**Figure 4:**
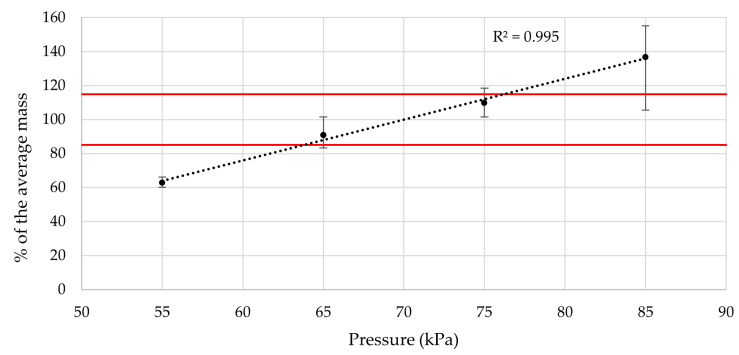
Average mass dependence on the pressure. Assuming the correlation between dose and tablet mass, the red lines represent the acceptable dose deviation limits of 85% and 115%.

As for the tablet mass, the correlation between the air pressure and the average weight was observed. The air pressure therefore directly influences the drug dose, posing as a variable print parameter, though at the price of some geometrical inaccuracy of product. Robustness-wise, assuming the ideal correlation between the average tablet weight and drug dose, variances in air pressure of approximately ±6 kPa should not cause a content to deviate over Ph. Eur. 10.0, Chapter 2.9.6. limits (85–115% drug content—highlighted by the red lines in the chart) [45]. Discussed data are presented in Figure 4.

The study by Díaz-Torres et al. (2022) used a different approach, evaluating the pressure created by applying differential flow rate, temperature, and nozzle diameter on three different materials. For this purpose, they incorporated the pressure sensor in their SSE 3D printer. They obtained results that suggest the printing speed of approximately 20 mm/s is the most suitable for printing the chosen gel-like formulation [51]. The extruder employed was, however, controlled mechanically, and such control is not available for pneumatically driven extrusion.

### 3.4. Texture Properties

To ensure the production of chewable gummy tablets of satisfactory quality, it is essential to avoid defects such as ruptures or holes, as these can adversely affect the mechanical properties of the tablets, potentially leading to disintegration upon handling. Rupture is defined as visible damage on the tablet, excluding microscopic damage [52]. Additionally, texture properties play a crucial role in patient acceptance and compliance with treatment [53].

In this study, two cycles of compression were employed to simulate the effects of chewing during the first few bites, offering insight into how the medicine behaves inside the mouth [54]. The primary focus was on tablet hardness (resistance against compression) and springiness.

The springiness index is a key indicator of an object’s ability to regain its original shape after compression. It is calculated by dividing the compressed height of the tablet by its original height. Ideally, an elastic object should return to its original height after one cycle of compression, corresponding to a springiness index of 1. A springiness index of 1 indicates 100% shape regeneration, while a value approaching 0 suggests irreversible deformation.

Since there is no pharmacopeial limit set for textural properties, well-known chewable gummy bears (Haribo^®^, Grafschaft, Germany) cut to uniform height of 4.5 mm were employed as a suitable standard. The examined properties were the resistance against compression and springiness index pointing at its elasticity and ability to sustain shape and size upon compression. A full overview of measured data may be found in Appendix C Table A2.

To analyze the results and determine whether there were significant differences between printed batches, one-way ANOVA was conducted. Differences with a probability level *p* < 0.001 were considered very significant, between *p* = 0.001 and 0.05 significant, and *p* > 0.05 not significant. The significance level (α) was set to 0.05 [55].

During the optimization phase, batches P5,5G11 and P6G10 showed the most similarity to the reference sample in terms of resistance against compression and springiness index. Due to slightly easier preparation, the P6G10 printing mixture was selected for further testing. Other batches exhibited lower springiness index values, indicating reduced elasticity. The differences in both hardness and springiness index between batches were considered very significant, with *p* values of less than 0.001 in both cases.

The addition of other components, including sweetener, colorant, antimicrobial agent, and flavoring agents, significantly affected (compared to P6G10 sample) springiness index (*p* = 0.001–0.05) and the positive impact on its resistance against compression was highly significant (*p* < 0.001).

The incorporation of ondansetron hydrochloride dihydrate did not negatively affect the compression resistance or springiness index of the tablets. However, a clear correlation was observed between model size and resistance to compression, with *p* < 0.001 indicating a highly significant relationship. Compression resistance values ranged from 1.87 to 9.60 N (the lowest corresponding to the 50% model size and the highest to 150%). In comparison, Adeleke and Abedin (2024) [56] reported values between 2.65 and 6.75 N when testing six different marketed gummy formulations. The slight discrepancy in values may be attributed to differences in product shapes, formulation compositions, and the size of the dosage forms. Our study also demonstrated that larger model sizes resulted in higher compression resistance. Additionally, the compression depth differed, with our study employing 2 mm, whereas Adeleke and Abedin employed 3 mm.

**Figure 5:**
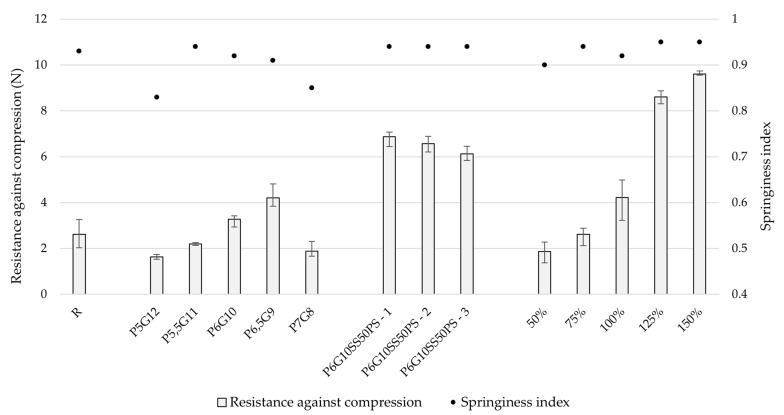
The selected texture properties of printed formulations.

As for the springiness, no direct correlation was observed between model size and springiness index, although differences between batches were considered very significant (*p* < 0.001). In the study by Adeleke and Abedin, the springiness index ranged from 0.96 to 1.00, while in this study, the final product exhibited a springiness index around 0.95, indicating satisfactory elasticity, which is desirable for this type of formulation [56]. The reference sample in our study showed a springiness index of 0.93, which closely matches the final product. Differences in these results can likely be attributed to variations in product shapes and formulations. All tested formulations included varying concentrations of gelatin as a gelling agent, which is commonly used in gummy preparations due to its positive impact on mouthfeel, chewing properties, and overall acceptability. The data are summarized in Figure 5.

### 3.5. Moisture Content

Moisture content is a critical factor influencing the biological stability and shelf life of chewable gummy tablets. Ideally, low moisture content is desired to maximize shelf life. However, excessively low moisture can increase the stiffness of certain formulations [57]. Fluctuations in moisture content can also degrade the quality of chewable gummy tablets by affecting properties such as stickiness and hardness, which may ultimately impact patient compliance [58,59]. All samples were compared to the reference sample, which exhibited a very low moisture content of 0.48%, with a relative standard deviation (RSD) of 2.08%. This can be attributed to the specialized process employed by Haribo^®^ to remove excess moisture.

During the optimization phase, the P6G10 sample demonstrated the lowest moisture content of 35.11%, making it a suitable composition for further use. Altering the pectin-to-gelatin ratio, either by increasing or decreasing pectin content, resulted in an increase in moisture content.

The addition of other excipients led to a decrease in moisture content, with an average of 26.42% across all three batches. This reduction was primarily due to the lower amount of water incorporated into the printing mixture compared to the optimization-phase samples.

Upon the incorporation of ondansetron hydrochloride dihydrate, a further decrease in moisture content was observed, ranging from 15.18% (50% model size) to 19.26% (75% model size). The variability in moisture content, as indicated by RSD, was satisfactory for most samples, closely matching the RSD of the reference sample. However, the 75% model size exhibited a higher RSD of 11.79%. These moisture content values were considerably higher than those reported in the study by Adeleke and Abedin (2024), which measured the moisture content of six marketed gummy formulations, ranging from 2.88% to 4.62% [56]. The discrepancy can likely be attributed to the differing compositions of the samples and the specific, extended drying process used for the commercially available products. The moisture content data are presented in Table 3.

Short-term stability studies lasting 5 and 10 days were conducted at 25 °C and 60% relative humidity to evaluate the final formulation’s tendency to absorb atmospheric moisture. Notably, a decrease in humidity levels was observed, indicating minimal risk of dosage form degradation due to excessive moisture uptake. As shown in Table 4, the moisture content closely aligns with the reference sample (0.48%), suggesting that the prepared dosage form is unlikely to degrade due to moisture accumulation over a short period. However, an increase in moisture content was observed between the 5th and 10th day of testing, with the most pronounced rise occurring in the largest model tablets, which displayed a twofold increase. This phenomenon may be attributed to their greater surface area, leading to increased moisture absorption capacity.

### 3.6. Repeatability Test

For the repeatability test, three batches of the same composition and model size were printed. These samples contained all ingredients except for ondansetron hydrochloride dihydrate, which was to be incorporated following the potentially successful repeatability testing. The focus was on assessing the variability of RSD through mass homogeneity testing, moisture content deviations between the batches, and variability in texture properties. In terms of mass homogeneity, all three samples complied with the requirements set by Ph. Eur. 10.0, Chapter 2.9.5. and RSD ranged from 1.35 to 2.49, which was in the context of compliance with the regulatory requirements considered satisfactory [45]. The springiness index was consistent across all three batches, with a value of 0.94, indicating no observed variability. Resistance against compression varied between 6.12 and 6.87 N, with deviations from the average ranging from 0.74 to 6.51%. These variations were within the acceptable range, and, compared to other batches with different compositions, the variability remained low. Moisture content ranged from 25.48 to 27.42%, with deviations from the average between 0.20 and 3.70%. This was in line with the requirements, as the moisture content showed minimal variation within the repeatability batches and was significantly different from that of other batches.

### 3.7. Disintegration Characteristics

Samples of all sizes containing the active ingredient were used for the disintegration test. Though there is no limit set in Ph. Eur. 10.0 for disintegration of chewable tablets, the possibility of a tablet being swallowed whole (intentionally or not) calls for the use of a 15 min limit specified for uncoated tablets [45]. All printed chewable gummy tablets exhibited satisfactory results as all of them disintegrated within 12 min. The relatively rapid disintegration (within 15 min) of gelatin-based chewable gummy tablets has been confirmed in several studies, indicating that this polymer is well suited for immediate-release formulations [60]. The selection of polymers in chewable gummy formulations plays a critical role in disintegration properties, as some formulations have been reported to exhibit significantly slower disintegration. Sabere et al. (2021) demonstrated that chewable gummy tablets containing agar and acacia gum either disintegrated very slowly (1.5 h) or failed to disintegrate entirely [61].

### 3.8. Drug Release

**Figure 6:**
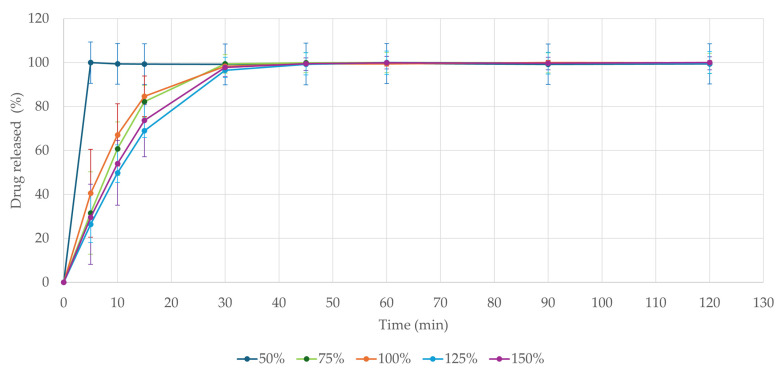
Dissolution profile comparison of prepared formulations.

The dissolution test is of high importance in peroral dosage forms. It provides crucial information and helps to predict drug absorption in vivo after administration [47,62]. As seen in Figure 6, all formulations released over 80% of the active ingredient within 30 min even when inserted in one piece. The fastest disintegration was observed in the 50% model-sized tablet, which rapidly disintegrated in the dissolution media, releasing the entire ondansetron load within 5 min. These results comply with the Ph. Eur. 10.0 Chapter 5.17.1. demands for conventional-release dosage forms (release of 80% of active substance in less than 45 min) [45]. The physical state of the dosage form plays a crucial role in its dissolution profile, though. In vivo fragmentation of the formulation through mastication has been shown to accelerate drug release by four times, as reported in the study by Sabere et al. (2021) [61]. Though tablets were tested in their intact form, they still classified as immediate-release formulations. Potential concerns regarding the dissolution profile of larger 3D-printed tablets could therefore be addressed through mastication or mechanical fragmentation.

**Figure 7:**
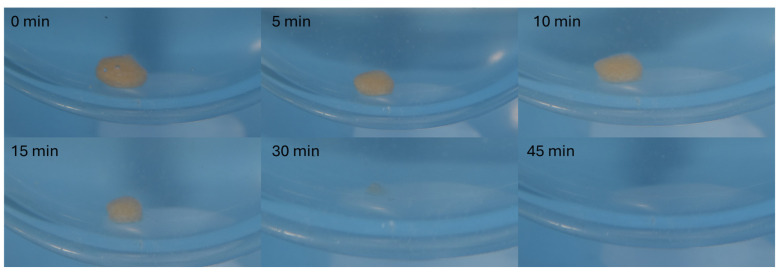
Dissolution of 150% sample at various time points.

All tablets fully dissolved within 30 min, as documented in Figure 7 on the largest model tablet (150%), releasing the full amount of the drug.

As suggested by Sabri et al. (2022) [63], the relatively rapid disintegration of the formulation may be attributed to the hydrophilic nature of pectin. Their study demonstrated that chewable gummy tablets primarily composed of pectin exhibited favorable dissolution characteristics, making them suitable as immediate-release formulations. Additionally, their findings indicate that reducing the pectin content to at least 4.5% could further enhance dissolution rates. Chewable gummy formulations based solely on gelatin [60] or its combination with hydroxypropyl methylcellulose (HPMC) [19] have also been reported to facilitate rapid drug release, typically within 30 min. Our findings further support the feasibility of developing chewable gummy tablets with an immediate-release profile.

To confirm the stability of the active ingredient in the dissolution media, four solutions of varying concentrations corresponding to 3, 4, 5, and 6 mg of ondansetron base in 900 mL of media were prepared and analyzed using the HPLC method described earlier in this article. The ondansetron content was measured at 30 min intervals for up to 3 h. The results (Table 5) indicate no concerns regarding the stability throughout the dissolution testing.

### 3.9. Ondansetron Content

The target dose for the base model size (labeled as 100%) was set at 4 mg, as discussed earlier in this article. To determine the amount of ondansetron hydrochloride dihydrate required, the addition was calculated based on the tablet’s average weight prior to incorporation of active substance. The tablet dimensions and its theoretical volume are summed up in Table 6.

The target dose of approximately 4 mg was successfully achieved with the base model size. Similar results were reported in the study carried out by Allahham et al. (2020), who printed orodispersible tablets containing ondansetron using selective laser sintering. In their study, they used HPLC to determine the ondansetron content, which was approximately 98.5% of the theoretical amount. Their findings indicated no degradation of ondansetron, even when utilizing a surface temperature of 100 °C and a chamber printing temperature of 80 °C [41]. The minor deviation in our results could be attributed to slight inconsistencies in the distribution of ondansetron hydrochloride dihydrate within the printing blend. Further testing involved changing the model size to 50%, 75%, 125%, and 150% of the original volume. The same printing mixture composition and settings were used to assess the influence of model size on drug content.

**Figure 8:**
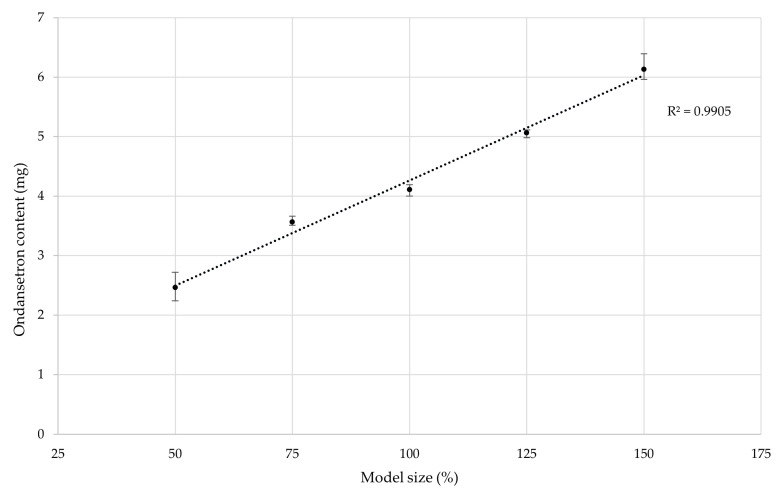
The correlation between the model size and drug content.

The correlation between the theoretical model size and the drug dose was demonstrated to follow a near-perfect linear regression. This confirmed that the drug dose directly correlates with the tablet’s weight, highlighting the importance of mass uniformity in minimizing drug content variability. The data are presented in Figure 8.

## 4. Conclusions

Chewable gummy tablets containing ondansetron hydrochloride dihydrate were successfully printed utilizing SSE 3D printing method. The tablets possessed satisfactory mechanical properties (comparable to the market standard for gummies) and complied with the requirements for the mass uniformity test according to Ph. Eur. 10.0, Chapter 2.9.5 [49]. While the moisture content was higher than that of the reference sample, this discrepancy can be attributed to the unique drying process used in its production, which was not replicated in this study as it prolongs the preparation. It was proven that the model size has a crucial impact on the tablet mass and the active ingredient content, with a linear correlation observed between the two. Larger model sizes resulted in higher resistance against compression.

In conclusion, a 4 mg dose of ondansetron was successfully approached with the base model, proving that SSE 3D printing method is a viable approach for producing chewable gummy tablets with individualized dosages. Additionally, the dissolution profile corresponding to conventional-release dosage form was proven in all model-sized formulations, confirming the suitability of the prepared chewable gummy tablets for immediate-release dosing. Further studies following the methodology of this study utilizing different active ingredients seem promising and represent the possible future direction of studies in the 3D printing field.

## Figures and Tables

**Table 1:** The composition of all print dispersion.

Composition Type	Sample	Excipient Concentration in Print Dispersion (wt.%)								
		P	G	PVP	CA	GLY	PS	SS	O	W
P to G ratio optimization	P5G12	5	12	5	2	20	-	-	-	Ad 100
	P5,5G11	5.5	11	5	2	20	-	-	-	Ad 100
	P6G10	6	10	5	2	20	-	-	-	Ad 100
	P6,5G9	6.5	9	5	2	20	-	-	-	Ad 100
	P7G8	7	8	5	2	20	-	-	-	Ad 100
PS and SS addition	P6G10SS50PS	6	10	5	2	20	0.3	28.35	-	Ad 100
O addition	P6G10SS50PS—O	6	10	5	2	20	0.3	27.05	1.2	Ad 100

P—pectin from apple, G—gelatin, PVP—polyvinylpyrrolidone, CA—citric acid, GLY—glycerol, PS—potassium sorbate, SS—simple syrup, O—ondansetron hydrochloride dihydrate, W—purified water.

**Table 2:** The dependance of weight on the theoretical volume of printed tablets.

Model	Volume (mm^3^)	Weight (mg)	Relative Weight (%)
50%	176.66	209.44	51.86
75%	265.06	312.14	77.29
100%	353.43	403.85	100.00
125%	441.76	493.61	122.23
150%	530.36	592.80	146.79

**Table 3:** The moisture content of printed formulations.

Composition Type	Sample	Moisture Content (%)	RSD
Reference sample	R	0.48 ± 0.01	2.08
P to G ratio optimization	P5G12	43.62 ± 0.90	2.06
	P5,5G11	38.07 ± 4.76	12.50
	P6G10	35.11 ± 1.65	4.70
	P6,5G9	43.73 ± 1.74	3.98
	P7G8	49.43 ± 2.20	4.45
PS and SS addition	P6G10SS50PS—1	26.37 ± 1.95	7.39
	P6G10SS50PS—2	25.48 ± 1.51	5.93
	P6G10SS50PS—3	27.42 ± 1.31	4.78
O addition	50%	15.18 ± 0.20	1.32
	75%	19.26 ± 2.27	11.79
	100%	19.14 ± 1.60	8.37
	125%	15.92 ± 0.17	1.07
	150%	17.44 ± 0.58	3.33

**Table 4:** Moisture content (%) in selected formulations after a specific period.

Sample	5 Days	10 Days	Change in % (5 vs. 10 Days)
50%	1.76 ± 0.30	2.09 ± 0.87	18.94
75%	1.94 ± 0.20	1.65 ± 0.15	−14.97
100%	1.78 ± 0.43	2.03 ± 0.27	14.07
125%	1.87 ± 0.69	2.06 ± 0.50	10.34
150%	2.35 ± 0.25	5.70 ± 6.02	142.07

**Table 5:** The concentration of ondansetron (mg/900 mL) in dissolution media in 30 min intervals.

Base Concentration	0 min	30 min	60 min	90 min	120 min	150 min	180 min	Change in % (0 vs. 180 min)
3	2.98	2.99	3.00	3.00	3.00	3.00	3.01	0.77
4	4.00	4.00	4.01	4.01	4.01	4.01	4.01	0.19
5	5.04	5.04	5.04	5.05	5.04	5.05	5.04	0.16
6	6.10	6.11	6.10	6.09	6.19	6.19	6.09	0.41

**Table 6:** The size of individual models. The volume represents a theoretical volume of the digital model. The name of the model then corresponds to relative volume.

Model	Diameter (mm)	Height (mm)	Volume (mm^3^)
50%	7.07	4.50	176.66
75%	8.66	4.50	265.06
100%	10.00	4.50	353.43
125%	11.18	4.50	441.76
150%	12.25	4.50	530.36

## Data Availability

Data are contained within the article.

## Abbreviations

The following abbreviations are used in this manuscript:

SSE	Semi-solid extrusion
CAD	Computer-aided design
HPMC	Hydroxypropyl methylcellulose

## Appendix A

**Table A1:** Currently marketed medicines with the content of ondansetron.

Brand Name	O Content	Excipients	Dosage Form	Patient Age	Manufacturer
Zofran	4 mg	Aspartam, methylparabenum, propylparabenum, propylenglyco, benzylalcohol, ethanol	Tablets	>6 months	Sandoz (Basel, Switzerland)
Setofilm	4 mg	polyvinyl alcohol, Macrogol 1000, acesulfam potassium, glycerol, titan dioxide, rice starch, levomenthol, polysorbate 80	ODFs	>4 years	LTS Lohmann Therapie-Systeme AG (Andernach, Germany)
Sigondan	4 mg	Lactose, microcrystallic cellulose, magnesium stearate, hypromellose, pre-gelatinized starch, titan dioxide, hyprolose, propylenglycol, sorbitan oleate, sorbic acid, vanillin,	Tablets	>6 months	Pharmathen S.A. (Marousi, Greece)
Ondansan	4 mg	Lactose, hydroxypropylcellulose, crospovidon, calcium silicate, aspartam, mint flavor, magnesium stearate, silicon dioxide,	ODTs	>6 months	
	4 mg	Natrium chloride, Natrium citrate-dihydrate, citric acid, aqua pro injectio	Ampules	>6 months	G. L. Pharma GmbH (Lannach, Austria)
	4 mg	Lactose, magnesium stearate, corn starch, microcrystallic cellulose, povidone, talcum, hypromellose, macrogol 6000, titan dioxide, iron oxide red	Tablets	>6 months	
Ondansetron Aristo	4 mg	Lactose, microcrystallic cellulose, pregelatinized starch, magnesium stearate, hypromellose, triacetin, titan dioxide, iron oxide yellow	Tablets	>6 months	Aristo Pharma GmbH (Berlin, Germany)

O—Ondansetron, ODFs—orodispersible films, ODTs—orodispersible tablets.

## Appendix B

TGA measurement of solvatomorphic state of used ondansetron hydrochloride was done on TGA7 (Perkin Elmer, Waltham, Massachusetts, USA) using nitrogen inert atmosphere. Sample was measured at the temperature slope of 10 °C/min in the range 50 to 290 °C. Obtained curve (Figure A1) shows loss of water by dehydration in the region from 20 to 140°C accounting to 10.02 wt.% loss, proving that ondansetron is in the dihydrate state.

## Appendix C

**Table A2:** Texture properties of prepared tablets and reference sample.

Composition Type	Sample	Hardness (N)	RSD	Springiness Index	RSD	Work (mJ)	RSD
Reference sample	R	2.61 ± 0.45	17.14	0.93 ± 0.02	2.15	1.90 ± 0.28	14.74
P to G ratio optimization	P5G12	1.64 ± 0.09	5.46	0.83 ± 0.03	3.61	1.24 ± 0.05	4.03
	P5,5G11	2.19 ± 0.05	2.37	0.94 ± 0.02	2.13	1.73 ± 0.04	2.31
	P6G10	3.27 ± 0.20	5.99	0.92 ± 0.02	2.17	2.38 ± 0.14	5.88
	P6,5G9	4.21 ± 0.49	11.54	0.91 ± 0.01	1.10	2.97 ± 0.23	7.74
	P7G8	1.88 ± 0.26	13.63	0.85 ± 0.04	4.71	1.51 ± 0.18	11.92
PS and SS addition	P6G10SS50PS—1	6.87 ± 0.25	3.65	0.94 ± 0.01	1.06	4.79 ± 0.15	3.13
	P6G10SS50PS—2	6.57 ± 0.29	4.42	0.94 ± 0.01	1.06	4.83 ± 0.21	4.34
	P6G10SS50PS—3	6.12 ± 0.22	3.56	0.94 ± 0.01	1.06	4.44 ± 0.11	2.48
O addition	50%	1.87 ± 0.40	21.25	0.90 ± 0.01	1.11	1.44 ± 0.28	19.44
	75%	2.61 ± 0.29	11.10	0.94 ± 0.01	1.06	2.03 ± 0.27	13.30
	100%	4.22 ± 0.71	16.82	0.92 ± 0.02	2.17	3.08 ± 0.54	17.53
	125%	8.61 ± 0.21	2.43	0.95 ± 0.01	1.05	6.31 ± 0.14	2.22
	150%	9.60 ± 0.07	0.77	0.95 ± 0.01	1.05	7.08 ± 0.05	0.71

P—pectin from apple, G—gelatin, PVP—polyvinylpyrrolidone, CA—citric acid, GLY—glycerol, PS—potassium sorbate, SS—simple syrup, O—ondansetron hydrochloride dihydrate, W—purified water.
